# Comparing Implementation Strategies for an Evidence-Based Weight Management Program Delivered in Community Mental Health Programs: Protocol for a Pilot Randomized Controlled Trial

**DOI:** 10.2196/45802

**Published:** 2023-05-10

**Authors:** Gerald J Jerome, Stacy Goldsholl, Arlene T Dalcin, Joseph V Gennusa 3rd, Christina T Yuan, Kristal Brown, Tyler Fink, Eva Minahan, Nae-Yuh Wang, Gail L Daumit, Kimberly Gudzune

**Affiliations:** 1 College of Health Professions Towson University Towson, MD United States; 2 Department of Medicine Johns Hopkins University School of Medicine Baltimore, MD United States; 3 Department of Health Policy and Management Johns Hopkins University Bloomberg School of Public Health Baltimore, MD United States; 4 Welch Center for Prevention, Epidemiology, and Clinical Research Johns Hopkins Medical Institution Baltimore, MD United States

**Keywords:** community mental health services, weight reduction programs, mental disorders, implementation science, mental health, randomized controlled trial, obesity, behavioral intervention, weight loss, strategy, community, weight, body mass index, BMI, obesity, mental illness, mental health, cardiovascular disease, psychiatric rehabilitation, RCT, coach, electronic coach

## Abstract

**Background:**

Among people with serious mental illness (SMI), obesity contributes to increased cardiovascular disease (CVD) risk. The Achieving Healthy Lifestyles in Psychiatric Rehabilitation (ACHIEVE) randomized controlled trial (RCT) demonstrated that a behavioral intervention tailored to the needs of individuals with SMI results in clinically significant weight loss. While the research team delivered the ACHIEVE intervention in the trial, community mental health program staff are needed to deliver sessions to make scale-up feasible. Therefore, we adapted the ACHIEVE-Dissemination (ACHIEVE-D) curriculum to ease adoption and implementation in this setting. Designing and testing of implementation strategies is now needed to understand how to support ACHIEVE-D delivery by community mental health program staff coaches.

**Objective:**

This study aims to conduct a pilot trial evaluating standard and enhanced implementation interventions to support the delivery of ACHIEVE-D in community mental health programs by examining effects on staff coaches’ knowledge, self-efficacy, and delivery fidelity of the curriculum. We will also examine the effects on outcomes among individuals with SMI taking part in the curriculum.

**Methods:**

The trial will be a cluster-randomized, 2-arm parallel pilot RCT comparing standard and enhanced implementation intervention at 6 months within community mental health programs. We will randomly assign programs to either the standard or enhanced implementation interventions. The standard intervention will combine multimodal training for coaches (real-time initial training via videoconference, ongoing virtual training, and web-based avatar-assisted motivational interviewing practice) with organizational strategy meetings to garner leadership support for implementation. The enhanced intervention will include all standard strategies, and the coaches will receive performance coaching. At each program, we will enroll staff to participate as coaches and clients with SMI to participate in the curriculum. Coaches will deliver the ACHIEVE-D curriculum to the clients with SMI. Primary outcomes will be coaches’ knowledge, self-efficacy, and fidelity to the ACHIEVE-D curriculum. We will also examine the acceptability, feasibility, and appropriateness of ACHIEVE-D and the implementation strategies. Secondary outcomes among individuals with SMI will be weight and self-reported lifestyle behaviors.

**Results:**

Data collection started in March 2021, with completion estimated in March 2023. We recruited 9 sites and a total of 20 staff coaches and 72 clients with SMI. The expected start of data analyses will occur in March 2023, with primary results submitted for publication in April 2023.

**Conclusions:**

Community mental health programs may be an ideal setting for implementing an evidence-based weight management curriculum for individuals with SMI. This pilot study will contribute knowledge about implementation strategies to support the community-based delivery of such programs, which may inform future research that definitively tests the implementation and dissemination of behavioral weight management programs.

**Trial Registration:**

ClinicalTrials.gov NCT03454997; https://clinicaltrials.gov/ct2/show/NCT03454997

**International Registered Report Identifier (IRRID):**

DERR1-10.2196/45802

## Introduction

The prevalence of obesity is significantly elevated in people with serious mental illness (SMI) [1,2]. Obesity is a leading cause of preventable death in this population, both directly through its effects on cardiovascular disease (CVD) and indirectly by contributing to other CVD risk factors [3-6]. In general populations, behavioral weight loss interventions have been demonstrated to improve CVD risk by reducing blood pressure and glucose, as well as improving lipid profiles [7]. However, behavioral interventions targeting changes in diet, exercise, and weight loss need to be tailored to the particular needs of people with SMI, such as memory impairment and limited executive function [8,9]. The National Institute of Mental Health (NIMH)–funded Achieving Healthy Lifestyles in Psychiatric Rehabilitation (ACHIEVE) randomized controlled trial (RCT) tested a behavioral weight-loss intervention for individuals with SMI who attended community-based psychiatric rehabilitation programs [10]. The ACHIEVE RCT demonstrated clinically and statistically significant weight loss [11]. ACHIEVE included group and individual weight-management sessions and group exercise sessions, which were led by trained study interventionists. Given the obesity epidemic in persons with SMI, there is an urgent need to disseminate ACHIEVE.

We posited that the ACHIEVE intervention might be ideal for dissemination by training and supporting community mental health program staff to deliver the sessions [12]. In the ACHIEVE RCT, the interventionists were study staff that received substantial onboarding and ongoing in-person training, as well as performance feedback with particular emphasis on motivational interviewing (MI) skills [10,11]. Time and other logistical constraints prevent community mental health program staff from participating in such intensive training. By leveraging novel strategies that incorporate advances in training and feedback modalities (eg, web-based training and web-based training environments), we could train community mental health program staff to facilitate the real-world implementation of the ACHIEVE program. We used the enhanced Replicating Effective Programs (REP) Framework for translating evidence-based interventions into community settings [13,14]. This model has 4 stages: preconditions, preimplementation, implementation, and maintenance. Preconditions identify needs, effective interventions, and implementation barriers to draft an intervention package. Preimplementation adapts and pilot tests implementation strategies for the community. Implementation focuses on training, support, evaluation, and refinement. Maintenance supports sustained implementation and dissemination.

We completed the REP preconditions stage for ACHIEVE by adapting the intervention to facilitate implementation by community mental health program staff while retaining core elements [15]. We modified the schedule and format to support efficient, staff-led delivery but retained the core messages and behavioral strategies. We previously evaluated this adapted curriculum in a proof-of-concept study at a community mental health program (NCT03999892) [16], which informed further refinements based on feedback from staff observers and participating individuals with SMI. Table 1 outlines this modified weight management curriculum, ACHIEVE-Dissemination (ACHIEVE-D), which will be used in the REP preimplementation stage.

**Table 1:** Overview of the ACHIEVE-Dissemination curriculum adapted for delivery by community mental health program staff.

		ACHIEVE-Dissemination curriculum^a^
Duration		6 months
**Group sessions**		
	1 group type	Multipurpose group weight management and exercise: 45-minute class, 3 times a week with weigh-ins (17 minutes of weight management, 20 minutes of moderate intensity exercise, and 8 minutes for weigh in)
Individual sessions		None
Total time		~540 minutes per month
Delivery modality of components		Weight management: led by staff member assisted with short videos and facilitator guide Exercise: video-assisted
Facilitators		Trained staff member and peer leader
**Behavioral messages**		
	6 messages	Weight loss success No sugar drinks No junk food Eat smart portions Eat more vegetables Putting it all together
Goal		5-lb weight loss in 6 months^b^ (tailored to individual)

^a^ACHIEVE: Achieving Healthy Lifestyles in Psychiatric Rehabilitation trial.

^b^Goal of a 5-lb weight loss at 6 months was based on results from the ACHIEVE trial [11] and adult obesity guidelines [7].

We describe the protocol for a pilot RCT comparing standard and enhanced implementation interventions that train and support community mental health program staff as coaches delivering the ACHIEVE-D weight management curriculum. This study represents our REP’s preimplementation stage. Our primary outcomes will be knowledge, self-efficacy, and delivery fidelity to the ACHIEVE-D curriculum among staff coaches. Both implementation interventions will include multimodal staff training and organizational strategy meetings. The staff coaches in the enhanced intervention will also receive performance coaching. We hypothesize that both implementation interventions will lead to increased knowledge of the ACHIEVE-D curriculum, self-efficacy for delivering the program, and fidelity for ACHIEVE-D delivery and that the enhanced intervention will have improved outcomes as compared to the standard intervention. To prepare for REP implementation and dissemination stages, we will use a mixed methods approach to examine acceptability, appropriateness, and feasibility, as well as compare differences in implementation and process measures to guide further refinements of both the ACHIEVE-D curriculum and implementation interventions. Secondarily, we will examine outcomes among individuals with SMI who participate in the curriculum, in particular self-reported lifestyle behaviors and measured weight.

## Methods

### Study Design

**Figure 1:**
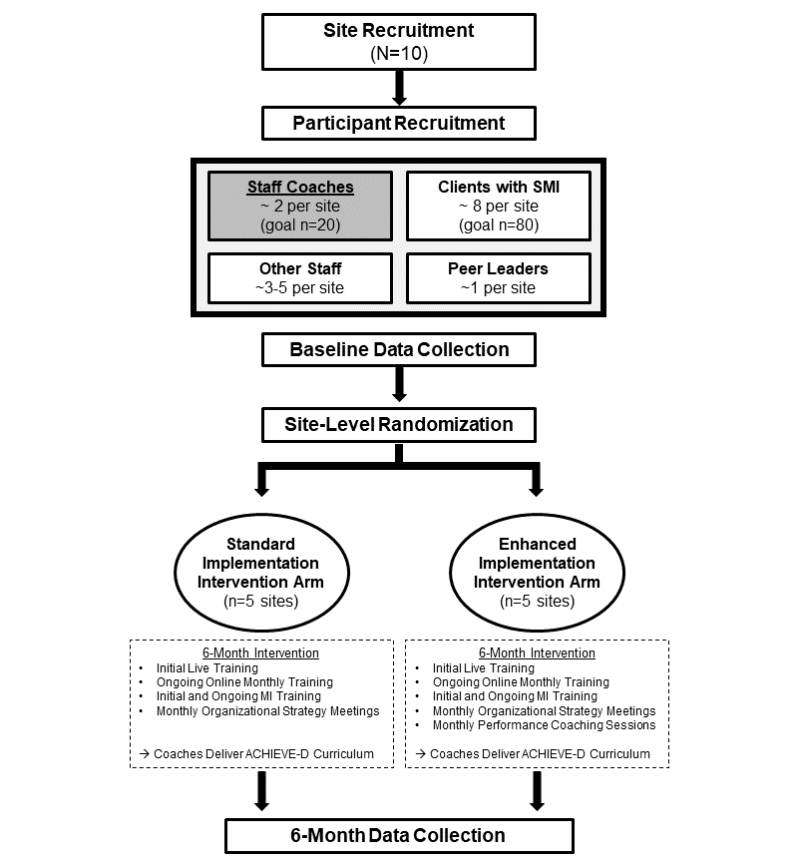
Overview of the study design. This diagram shows the planned study steps in this pilot randomized controlled trial. The gray shaded box indicates our primary study participant group who will take part in the implementation interventions. ACHIEVE-D: Achieving Healthy Lifestyles in Psychiatric Rehabilitation-Dissemination; SMI: serious mental illness.

The design will be a cluster-randomized, 2-arm parallel pilot RCT comparing standard and enhanced implementation intervention at 6 months within community mental health programs. We will aim to randomly assign 10 programs in Maryland to either the standard or enhanced implementation intervention arms. Figure 1 provides an overview of the study. Multimedia Appendix 1 contains the SPIRIT (Standard Protocol Items: Recommendations for Interventional Trials) checklist for RCTs [17].

### Overview of the ACHIEVE-D Curriculum

The ACHIEVE-D program is a 6-month curriculum comprised of structured group sessions incorporating behavioral weight management lessons along with group exercise. Groups will last approximately 45-60 minutes and occur at least once a week. Groups may be delivered in person or remotely. Table 1 outlines the components of the ACHIEVE-D curriculum, and Multimedia Appendix 2 includes a detailed description of the ACHIEVE-D curriculum. The curriculum has been previously tested in other studies [11,16]. As such, this study will focus on testing implementation interventions to support the delivery of the curriculum by community mental health program staff.

### Site Eligibility Criteria

We will aim to recruit 10 community mental health programs as study sites. Site inclusion criteria include (1) an adult psychiatric rehabilitation program (PRP) within Maryland, (2) a willingness for at least 2 staff to be trained as coaches and implement the ACHIEVE-D curriculum, (3) a willingness for leaders and staff to participate in monthly organizational strategy meetings, and (4) the anticipated ability to recruit clients with SMI to participate. Site leadership will indicate their agreement to fulfill these criteria with study staff and complete a reliance agreement for the study with the Johns Hopkins Institutional Review Board. At each site, we will recruit participants from three different study populations: (1) staff to serve as coaches; (2) clients with SMI to take part in the curriculum; and (3) other staff and peer leaders—we describe the eligibility criteria, recruitment, and measures separately for each group of study participants in the sections below.

### Coaches

#### Eligibility and Recruitment

To be included, the person must be ≥18 years old, staff at an enrolled PRP, and willing to complete all training activities, deliver all components of the ACHIEVE-D curriculum, and complete data collection procedures. We will exclude staff that plans on leaving their position or taking a leave of absence from the program. Site leadership will identify potential staff to serve as coaches, and the research team will approach these individuals to discuss the study and obtain a waiver of documentation of consent. We will aim to recruit 20 coaches (at least 2 per site).

#### Measures

Table 2 describes the assessment schedule for the primary outcomes and implementation measures for coaches. Multimedia Appendix 3 describes other measures that we plan to collect among coaches. The following will be the primary outcome and implementation measures for coaches.

**Table 2:** Assessment schedule for coaches: primary outcomes and implementation measures.

		Baseline	Follow-up		
			During	6M^a^	Post^b^
**Primary Outcomes**					
	ACHIEVE-D^c^ Curriculum Weight Management Knowledge	✓		✓	
	ACHIEVE-D Curriculum Exercise Knowledge	✓		✓	
	ACHIEVE-D Curriculum Self-Efficacy	✓		✓	
	ACHIEVE-D Fidelity		✓		
**Implementation Measures**					
	Acceptability of training	✓		✓	
	Feasibility of training	✓		✓	
	Appropriateness of training	✓		✓	
	Motivation for implementation	✓		✓	
	Implementation climate	✓			
	Focus groups or interviews	2		✓	✓

^a^M: month.

^b^Postperiod will be approximately 6 months after completion of the intervention.

^c^ACHIEVE-D: Achieving Healthy Lifestyles in Psychiatric Rehabilitation-Dissemination.

### Primary Outcomes

#### ACHIEVE-D Curriculum Knowledge, Self-Efficacy, and Fidelity

We will assess knowledge about key curricular elements using 2 measures at baseline and 6 months: a 22-item ACHIEVE-D Curriculum Weight Management Knowledge measure and a 16-item ACHIEVE-D Curriculum Exercise Knowledge measure. The research team created these questions using the best practices recommended by the National Board of Medical Examiners for item writing [18]. We will assess self-efficacy to deliver key curricular elements at baseline and 6 months using a 31-item ACHIEVE-D Curriculum Self-Efficacy measure. The research team used principles from confidence rulers in creating this measure [19], where participants rate their confidence to deliver each element on a 10-point scale. To determine fidelity, each coach will video-record their delivery of an ACHIEVE-D group session once a month. Two research team members will view each recording and use the ACHIEVE-D Curriculum Fidelity rating tool to assess performance on key elements. The research team created this tool to evaluate how a coach implements key ACHIEVE-D curriculum elements, including a counseling style consistent with MI techniques (eg, open-ended questions and affirmations) [20], within each segment (before the session, group exercise, weigh-in, focus on weight loss, role model video, commitment to high-impact behavior). Each item will be scored as absent (0) or present (2) or on a 3-point scale of absent (0), below expectations (1), or meets expectations (2). Quarterly meetings will be conducted to ensure alignment between coders. Table 3 displays example items from instruments used for the primary outcomes.

**Table 3:** Samples of included items in primary outcome measures.

Primary outcomes		Sample questionnaire items
**ACHIEVE-D^a^ Curriculum Weight Management Knowledge**		
	Example 1	An evidence-based weight loss program includes which of the following? Uses solid scientific evidence to drive recommendations^b^ Promises results that sound too good to be true Has evidence from personal testimonials about weight loss success Don’t know
	Example 2	Jessica is taking part in ACHIEVE. She wants to say “no” to junk foods, but needs help identifying foods that she eats that are considered junk foods. Which of the following things that she eats would be considered a junk food in the ACHIEVE program? Orange slices Chocolate bars^b^ Cheese Don’t know
**ACHIEVE-D Curriculum Exercise Knowledge**		
	Example 1	What should you do if a client has arthritis in their knees and cannot keep up with the class choreography? Tell the client with the arthritis that they must do a different exercise move Choose slower music for the entire class so that all clients can keep up Remind all clients that they can choose a lower intensity^b^ Don’t know
	Example 2	Joy is 45 year old woman with obesity. She is sedentary (does not exercise). If Joy starts going for a 20 minute brisk walk every morning, she will: Start losing weight Will not lose weight, because exercise doesn’t help with weight loss May not lose weight, but will get benefit from exercising^b^ Don’t know
**ACHIEVE-D Curriculum Self-Efficacy^c^**		
	Example 1	Use a leader’s guide along with any other needed materials, props, visual aids, videos, or lesson presentations for the class. 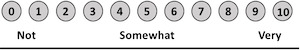
	Example 2	Communicate with clients how their diet, activity and other behaviors aligns with weight change. 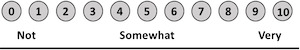
**ACHIEVE-D Curriculum Fidelity^d^**		
	Example 1	Asks about success with high impact behavior. 0: Does not ask participants about progress on high impact behavior 1: Asks some participants about progress on high impact behavior 2: Asks all participants about progress on high impact behavior
	Example 2	Ensures session remains focused on main topic as per leaders guide. 0: Did not focus on the main topics in the leaders guide for this module 1: Some topic elements covered, but a major deviation from a main topic occurred 2: All topic elements covered with minor acceptable deviations

^a^ACHIEVE-D: Achieving Healthy Lifestyles in Psychiatric Rehabilitation-Dissemination.

^b^Indicates correct responses. Complete measures are available from the authors upon request.

^c^Participants indicate how confident they are that they can currently perform the listed task during a behavioral weight loss program for a group of adults with serious mental illness.

^d^All items have a numerical value, and we will sum key items to create a composite score.

#### Implementation Measures

We will determine the perceived acceptability, feasibility, and appropriateness of the implementation interventions (eg, coach training) and the ACHIEVE-D curriculum. We adapted these assessments from previously validated measures [21]. We will also assess motivation and implementation climate using adapted, validated instruments [22,23]. At 6 months, we will conduct focus groups with staff coaches to elicit feedback on the following: acceptability, appropriateness, and feasibility of the implementation intervention and ACHIEVE-D curriculum; barriers to implementation; strategies to overcome barriers; and suggestions for further refinement. We will conduct a second series of focus groups or interviews at each site approximately 6 months after the conclusion of the intervention to determine whether and how the ACHIEVE-D curriculum was sustained and the challenges experienced.

### Clients With SMI

#### Eligibility and Recruitment

Clients with SMI will participate in the ACHIEVE-D weight management program delivered by the staff coaches. To be included, the person must be ≥18 years old, a client with SMI at an enrolled PRP, BMI ≥25 kg/m^2^ (ie, overweight or greater), and willing to make lifestyle changes to lose weight and participate in all ACHIEVE-D activities at least once a week. We will exclude individuals with contraindications to losing weight (eg, history of anorexia, liver failure, and pregnancy), who have conditions that require medically supervised weight loss (eg, myocardial infarction in the last 6 months and insulin-dependent diabetes mellitus), who plan to pursue other obesity treatments (eg, antiobesity medication and bariatric surgery), who plan to leave the PRP or move out of the geographic area within 12 months, or who are unwilling to complete all data collection procedures. Staff at each site will identify potential clients to participate who may meet the eligibility criteria; the research team will approach these individuals, on a first-come, first-served basis, to discuss the study, screen individuals, and obtain a waiver of documentation of consent. We will aim to recruit 80 clients with SMI (~8 per site).

#### Measures

Table 4 describes the assessment schedule for individuals with SMI. Multimedia Appendix 3 describes other measures that we plan to collect among individuals with SMI. In other studies, we have successfully used all selected measures among individuals with SMI. Similar to our prior studies, study staff will be available to assist clients with SMI complete measures, if needed. The following will be the secondary outcome and process measures for individuals with SMI.

**Table 4:** Assessment schedule for individuals with serious mental illness: secondary outcomes and process measures.

		Baseline	Follow-up	
			During	6M^a^
**Secondary outcomes**				
	Weight^b^	✓		✓
	CARDIA^c^/EARLY^d^ Q^e^—sedentary behavior	✓		✓
	Block Dietary Fat Q	✓		✓
	Block Dietary Fruit or Vegetable Q	✓		✓
	EARLY Eating Away from Home Q	✓		✓
	EARLY SSB^f^ Consumption Q	✓		✓
**Process measures**				
	Consumer Satisfaction and Helpfulness Q			✓
	Attendance		✓	
	Participation in group session		✓	

^a^M: month.

^b^If weight measurement cannot be done in person, due to COVID-19 restrictions, participants will self-measure weight on a scale that the study provides to them.

^c^CARDIA: Coronary Artery Risk Development in Young Adults.

^d^EARLY: Early Adult Reduction of Weight through Lifestyle Intervention.

^e^Q: questionnaire.

^f^SSB: sugar-sweetened beverage.

### Secondary Outcomes

#### Weight and Lifestyle Behaviors

Study staff will measure weight to the nearest 0.1 lb. on a high-quality digital scale with participants wearing light indoor clothes without shoes at baseline and 6 months. We will assess several lifestyle behaviors at baseline and 6 months. Study staff will administer the Coronary Artery Risk Development in Young Adults (CARDIA)–Early Adult Reduction of Weight through Lifestyle Intervention (EARLY) sedentary behavior questionnaire [24]. For diet, we will administer the block fat, fruit, vegetable, and fiber screener questionnaires [25,26]. We will also administer the EARLY eating away from home and sugar-sweetened beverage questionnaires [27,28]. These measures have been recommended by the National Institutes of Health Accumulating Data to Optimally Predict Obesity Treatment (ADOPT) Core Measures for Obesity Studies [29].

#### Process Measures

We will adapt prior measures to assess the helpfulness of the behavioral weight loss intervention component [30] and satisfaction with the ACHIEVE-D curriculum and staff coaches [31]. We will collect attendance and participation information for each enrolled client.

### Other Staff: Eligibility, Recruitment, and Measures

We will recruit 2 groups of other staff: program leaders (ie, program directors or coach supervisors) and general staff members. Program leaders will participate in monthly organizational strategy meetings with the coaches. General staff members will not participate in training or meetings. To be eligible as other staff, the person must be ≥18 years old, staff at an enrolled PRP, and willing to complete data collection procedures. After site leadership facilitates contact with other staff, the research team will approach these individuals to discuss the study and obtain a waiver of documentation of consent. All other staff will complete baseline demographics as well as motivation for implementation and implementation climate [22,23] measures at the baseline and 6-month follow-up. Program leaders will be in the aforementioned end-of-study focus groups with the coaches.

### Peer Leaders: Eligibility, Recruitment, and Measures

When possible, we will recruit a peer leader for each site. To be eligible, the individual must be a person with SMI who is ≥18 years and interested in helping staff coaches with the ACHIEVE-D curriculum. Staff at each site will identify potential clients to participate as peer leaders; the research team will approach these individuals to discuss the study and obtain a waiver of documentation of consent. The study will proceed at sites without peer leader support if no candidates are identified. Peer leaders will participate in the following: training with the staff coaches, assisting staff coaches in the delivery of the ACHIEVE-D curriculum to clients, modeling behavior by participating in group exercises, and completing data collection (same measures as coaches except for ACHIEVE-D curriculum fidelity).

### Implementation Interventions

#### Overview

The study team’s prior research and experience with ACHIEVE informed our selection of components that will comprise the implementation interventions for the standard and enhanced arms. Both arms will use multimodal training in combination with organizational strategy meetings with site leadership to support the implementation of the ACHIEVE-D group sessions. In the enhanced arm, staff coaches will also receive performance coaching from research team members with expertise in weight management and MI.

#### Multimodal Coach Training—Both Arms

Table 5 provides an overview of all coach training that will be conducted by the research team during the 6-month intervention period. Multimodal training will include real-time initial training via videoconference, ongoing monthly virtual training, and web-based avatar-assisted MI practice for coaches [32,33]. This training approach will provide interactive opportunities for coaches to practice the skills needed to implement the ACHIEVE-D curriculum—a more effective strategy than didactic education [34]. All coaches will receive initial training in real-time delivered via videoconferencing (approximately 14 hours in total). The initial training will include an introduction to MI, highlighting the effectiveness of this counseling style for weight management. Ongoing monthly training will be offered through a digital platform with the goal of orienting coaches to upcoming content (less than 30 minutes per month). The coaches will also have access to a web-based avatar module to review and practice MI techniques when discussing weight management with a simulated client avatar. The research team developed this module in partnership with a health care simulation company. Coaches will receive reminders to complete the monthly web-based training and avatar modules.

**Table 5:** Overview of ACHIEVE-Dissemination trainings for coaches.

Type and training components		Approximate length
**Initial training (synchronous, videoconference, and research team–delivered)**		
	Overview of the ACHIEVE^a^ program and introduction to behavior change for weight loss	2 hours
	Components of an ACHIEVE group and observing research staff deliver a group lesson	2 hours
	Introduction to Module 1: Weight Loss Success	45 minutes
	Weight management practice session	60 minutes
	MI^b^ techniques for weight loss	4 hours
	Common challenges that may come up when running a weight loss group and how to address them	60 minutes
	Exercise in the ACHIEVE program	20-60 minutes
	Introduction to Module 2: Say No to Sugar Drinks	50 minutes
	Resources for implementing the ACHIEVE program	20 minutes
	Overview of performance coaching	40 minutes
**Ongoing training (asynchronous, digital, and self-paced)**		
	Didactic training for each core and seasonal module with real-world examples of how to deliver the content in groups	15-20 minutes per month
	Avatar simulations to practice using motivational interviewing skills when discussing weight management with a consumer	15 minutes per month

^a^ACHIEVE: Achieving Healthy Lifestyles in Psychiatric Rehabilitation.

^b^MI: motivational interviewing.

#### Organizational Strategy Meetings—Both Arms

Programs targeting frontline staff, like ACHIEVE-D, are more effective when supplemented with implementation interventions that successfully engage organization leaders [35]. Therefore, to enhance curriculum uptake and increase engagement from site leadership [36], we will hold monthly organizational strategy meetings (30-60 minutes each month). Research team members, coaches, and site supervisors or leadership will be encouraged to attend. These meetings will support the development, adoption, and sustainment of organizational strategies needed for implementation and provide opportunities to problem-solve barriers encountered (eg, changing the class schedule to maximize attendance).

#### Performance Coaching—Enhanced Arm Only

At sites randomized to the enhanced intervention arm, coaches will take part in monthly performance coaching. Performance coaching is an implementation strategy where coaches “learn by doing” and receive feedback on their ACHIEVE-D delivery [37]. Performance coaching may be particularly beneficial when evidence-based practice is new to the implementers, as is the case with ACHIEVE-D [38-40]. The performance coach will be a research team member with extensive knowledge of the ACHIEVE-D curriculum, behavioral weight loss, and MI. Each month, the performance coach and staff coach will meet to review the staff coach’s monthly video-recorded group session (see the video recording described in coach measures above). The performance coach will share their evaluation of the video, identifying areas of strength, opportunities for improvement, and a performance improvement plan (1.5 hours each month). Performance coaching feedback will be grounded in evidence-based behavioral weight loss principles and core ACHIEVE-D messages, mirror a MI style, and promote skill development and high-fidelity curriculum delivery. Of note, the study staffs who deliver performance coaching will be different from the study staff rating the video-recorded sessions for fidelity.

### Pilot Trial Sample Size

This pilot trial will provide useful information on protocol feasibility and parameter estimates for designing a future confirmatory trial but will not necessarily be powered to detect differences in outcomes. With 5 sites per arm and 2 coaches per site, we will have 80% power to detect large changes in effect size in our primary coach-level outcomes from baseline to 6 months between arms (1.5-1.6 in units of outcome SD). We assume an intracluster correlation coefficient of 0.05-0.2 and a within-coach correlation of 0.5 between outcomes at baseline and 6 months, using a 2-sided alpha of .05.

### Randomization and Blinding

Following recruitment and baseline data collection, sites will be randomized to the standard or enhanced arm. The randomization sequence will be generated in the block size of 2 using computer software by the trial biostatistician, and assignments will be delivered to the eligible sites by the research coordinator. Due to the nature of the intervention, both staff coaches and some research team members will be aware of the assignment. For our primary outcomes of knowledge and self-efficacy, staff coaches will self-complete specific questionnaires, which may reduce the risk of bias. For our primary outcome of fidelity, study team members who rate the video-recorded sessions will be masked to study arm assignment, which may also reduce the risk of bias. Individuals with SMI taking part in the curriculum and other staff will not be aware of the assignment.

### Statistical Analysis

#### Quantitative Analyses

We will assess the effects of the implementation intervention on coaches’ knowledge, self-efficacy, and fidelity to the ACHIEVE-D curriculum, as well as client outcomes, using generalized linear mixed-effects models under the intent-to-treat approach. The mean models will include a binary intervention group indicator, time indicators, and intervention-by-time interaction terms. The time indicator coefficients estimate time-specific mean outcome changes in the standard arm, and the interaction coefficients estimate the between-arm differences of time-specific outcome changes from the baseline. The linear combination of these time-specific coefficients captures the time-specific mean outcome changes in the enhanced arm. Site differences will be assumed to follow a normally distributed random effect, while an unstructured variance-covariance matrix will be used for the repeated outcome measurements over time within individuals. For our implementation measures, we will use descriptive statistics to analyze survey measures of perceptions of acceptability, feasibility, and appropriateness of the implementation intervention and ACHIEVE-D curriculum. Point estimates and corresponding 95% confidence intervals will be obtained.

#### Qualitative Analyses

Focus groups will be audio- or video-recorded, and recordings will be transcribed. Given that we are seeking to understand experiences across sites and participants, we will use thematic analysis [41,42], which can serve as a standalone analytic method. We will use a 6-step process, which includes data familiarization, initial code generation, searching for themes (ie, repeated patterns), reviewing themes, defining or naming themes, and producing a report. The first 3 steps will be conducted by 2 research team members, and the larger research team will be involved in the final steps.

### Ethics Approval

The Johns Hopkins University School of Medicine Institutional Review Board approved this study (IRB00247344). Consent will be obtained from all participants using a waiver of documentation of consent.

## Results

This trial was funded by an ALACRITY center grant from the NIMH (funding started in August 2018). We started recruitment and data collection in March 2021. We estimate all data collection will be completed in March 2023. We recruited 9 sites and a total of 20 staff coaches and 72 clients with SMI. We expect to start data analyses in March 2023, with primary results submitted for publication in April 2023. We will also communicate the results with participating sites after the study through a presentation with leadership or a report.

## Discussion

### Principal Findings

Addressing inequities in CVD risk among persons with SMI is needed to meet current public health objectives of improving cardiovascular health for all persons [43]. Given the prevalence of obesity among adults with SMI and its associated increased CVD risk, it is critical to implement effective behavioral weight loss interventions tailored to this population in real-world settings. The ACHIEVE trial demonstrated the efficacy and safety of such a behavioral weight loss intervention [11], which has been translated into the ACHIEVE-D weight management curriculum appropriate for delivery by community mental health center staff [16]. We are applying the Enhanced Replicating Effective Programs Framework to increase the acceptability and feasibility of implementing the ACHIEVE-D curriculum in community settings [13,14]. In this pilot trial, we will compare an enhanced implementation intervention to a standard intervention on community mental health program staff coaches’ knowledge, self-efficacy, and fidelity to the ACHIEVE-D curriculum.

We will use a mixed methods approach [44], where the design will be primarily quantitative with a sequential supplementary qualitative component among staff coaches. The quantitative component will determine whether the staff coaching approach is a feasible one, while the qualitative component will be key to understanding staff experiences as coaches and leadership experiences with having ACHIEVE-D at their site. We anticipate that we will identify patterns of facilitators and barriers common across staff and sites through the focus groups, and we will use these findings to guide further refinements in the ACHIEVE-D curriculum and implementation interventions.

There are several potential strengths in this study. We are training community mental health program staff to deliver the curriculum, which may have numerous advantages when considering dissemination. Staff members already have an established rapport with clients, which may be helpful when addressing sensitive topics such as weight. The ACHIEVE-D curriculum is a turnkey product with coach guides and videos for staff to use, which may minimize work burden and increase confidence in providing safe, evidence-based recommendations to clients. In addition, we created an adapted and abbreviated strategy for remotely delivered training to program staff, which may address potential time and logistical constraints [12]. Asynchronous web-based training will be brief, which may enhance coach uptake and completion of this training as this approach meets the needs of staff with competing priorities. Since training alone is often insufficient to adopt new interventions [45-48], this trial will incorporate organizational strategy meetings to engage leadership and test the addition of performance coaching in the enhanced arm. Finally, we anticipate recruiting diverse program staff with varying years of experience, education, and profession (eg, counselors and nurses) and diverse organizations with varying staffing models, geographic locations (eg, urban, suburban, and rural), and delivery models (eg, in-person and remote). This diversity may provide novel lessons relevant for dissemination. Overall, these strengths may help support testing of future REP stages of implementation and maintenance of the ACHIEVE-D curriculum in community mental health settings through a hybrid effectiveness-implementation trial approach [49].

### Limitations

Our pilot trial will have limitations and challenges. Our study sites will be limited to community mental health programs in Maryland, specifically psychiatric rehabilitation programs. Other states may not offer such programming, and billing practices may differ, which may limit generalizability. This trial may be impacted by the high staff turnover rates that are common in community mental health programs. In anticipation of this challenge, we plan to recruit at least 2 staff coaches for each site and have the flexibility to train replacement coaches if needed. This study is a pilot trial, and as such, our sample size is small. The COVID-19 pandemic will present several challenges, as this trial will be recruiting and conducting the study during this period. First, community mental health program staff turnover may be even higher, which may negatively impact staff engagement in the study due to increased workload. Second, PRP may experience changes related to COVID-19 restrictions, such as format (in-person versus remote) and the number of clients permitted on-site. These factors may affect delivery and client attendance at ACHIEVE-D sessions. Third, sites might experience closures due to COVID-19, which would result in gaps in ACHIEVE-D delivery. As consistency and repetition are key elements for clients with SMI, such gaps may affect client engagement and participation, as well as weight and behavioral outcomes. Finally, we will be unable to blind coach participants or interventionists to study arm allocation due to the nature of the intervention in this pilot trial; however, we will mask the study assignment of study team members to determine fidelity. Future studies should consider blinding data collectors and analysts to study arm allocation.

### Conclusions

This trial aims to provide key information on implementation strategies to increase community mental health program staff coaches’ knowledge, self-efficacy, and fidelity to deliver the ACHIEVE-D weight management curriculum for persons with SMI. Our results may inform future research that definitively tests the implementation and dissemination of behavioral weight management programs. Unless effective weight management interventions are disseminated into real-world settings, obesity and CVD disparities among persons with SMI will likely persist.

## Appendix

Multimedia Appendix 1 SPIRIT (Standard Protocol Items: Recommendations for Interventional Trials) checklist and figures.

Multimedia Appendix 2 Detailed information about Achieving Healthy Lifestyles in Psychiatric Rehabilitation-Dissemination (ACHIEVE-D) curriculum.

Multimedia Appendix 3 Other planned measures for coaches and individuals with serious mental illness.

## Abbreviations

ACHIEVE: Achieving Healthy Lifestyles in Psychiatric Rehabilitation
ACHIEVE-D: Achieving Healthy Lifestyles in Psychiatric Rehabilitation-Dissemination
CVD: cardiovascular disease
MI: motivational interviewing
PRP: psychiatric rehabilitation program
RCT: randomized controlled trial
REP: replicating effective programs
SMI: serious mental illness

## Data Availability

Data sharing is not applicable to this paper as no data sets were generated or analyzed during this study. Future data collected as part of the study described in this protocol will be made available from the corresponding author upon reasonable request.
