# Supplementary material for: Comparing Implementation Strategies for an Evidence-Based Weight Management Program Delivered in Community Mental Health Programs: Protocol for a Pilot Randomized Controlled Trial
Source: JMIR Res Protoc. 2023 May 10;12:e45802. doi: 10.2196/45802 (PMC10209790; doi:10.2196/45802)
Supplement: Multimedia Appendix 2 [file resprot_v12i1e45802_app2.docx]

**Multimedia Appendix 2: Detailed Information about ACHIEVE-D Curriculum**

Obesity is a chronic condition that benefits from ongoing intervention, and we designed the ACHIEVE-Dissemination (ACHIEVE-D) curriculum to be integrated into the regular programming schedule at each site. The curriculum was adapted from the previously tested behavioral intervention in the ACHIEVE randomized controlled trial,^[[1]](#footnote-1)^ which demonstrated its efficacy and safety. Our team also previously tested two months of the adapted ACHIEVE-D curriculum at one community mental heath program in a proof-of-concept study, which found that the format was feasible and acceptable to clients with serious mental illness (SMI) and program staff.

*Theoretical Framework for ACHIEVE-D Curriculum.* The theoretical framework of the ACHIEVE-D curriculum incorporates social cognitive and behavioral self-management theories and is congruent with psychiatric rehabilitation principles of skill building and environmental supports.^[[2]](#footnote-2),^^[[3]](#footnote-3),^^[[4]](#footnote-4)^ The curriculum also encourages using a Motivational Interviewing communication approach and builds on behavioral lifestyle interventions that have shown to be effective in the general population.^[[5]](#footnote-5),^^[[6]](#footnote-6)^

*Curriculum Tailoring for Individuals with SMI and Community Mental Health Program Setting.* The ACHIEVE-D curriculum was tailored to address deficits in memory and executive function that are common among individuals with SMI by incorporating simplified messaging, repetition of key program elements, and dividing information into small components. This strategy was shown to be effective in the ACHIEVE trial.^1^ In addition, an overarching principle of the curriculum design is fit into the regular programming that community mental health programs are already providing for their clients with mental illness. The program staff trained as coaches will also have the support of their leadership for ACHIEVE-D curriculum delivery (e.g., given time to complete trainings, prepare for groups, and complete study documentation).

*Overview of ACHIEVE-D Curriculum.* All sites will use the ACHIEVE-D curriculum – a 6-month behavioral weight-loss program delivered by trained staff coaches to enrolled clients with SMI at 10 community mental health programs. Coaches will deliver multipurpose ACHIEVE-D group sessions, which include weight management along with group exercise. Each group session lasts between 50-60 minutes. Coaches will be provided with resources to deliver the group sessions including role model videos to be used in class to reinforce the primary weight loss strategies, group exercise videos, as well as all required materials.

*Curriculum Materials.* We have developed a turn-key product for community mental health program staff to implement the ACHIEVE-D program at their sites. Included are a *Coach Implementation Guide* that provides an overview of the program and a description of each component of group. *Leader’s Guides* for each lesson include a list of objectives and materials (e.g., posters, scale, handouts) needed and provide sample language for how the content might be delivered. Digital copies of the manual, guides, and handouts will be available to coaches online.

*Group Session Frequency & Delivery Modality.* ACHIEVE-D group sessions will be offered at least once per week and up to three times per week. Group sessions may be delivered either in-person, remotely (via videoconference), or a combination of both. Delivery modality is tailored to the needs of each site – factors that may influence modality used includes site capacity, staffing, billing considerations, as well as COVID-19 restrictions. When group sessions are offered remotely, the study will provide enrolled clients with tablets that have data plans to facilitate their participation from home (at no cost to the participant). Coaches will also receive technology support from a research team member who will manage the technical aspect of the videoconference platform including launching exercise and role model videos, displaying the lesson’s digital posters, and assisting with study documentation. An overview of the ACHIEVE-D curriculum can be found in Supplementary Table 1.

*Structure of Group Session.* Coaches will use Leader’s Guides for each lesson and all lessons follow a similar structure. Each lesson starts with an exercise component, which is then followed by weigh-in, weight management lesson, and goal setting.

*Exercise Component – 20 Minutes.* Groups include video-led group exercise at a level appropriate for sedentary persons, with gradual increases in duration and intensity.^[[7]](#footnote-7)^ Videos will include low-impact, moderate intensity, aerobic exercise; coaches will monitor clients for safety and provide individualized cueing to ensure they are exercising at an appropriate intensity. Each exercise session will include both a warm up and cool down. Exercise classes may be delivered via Zoom video-conferencing as part of remote groups. In-person group exercise classes may be offered during COVID-19 with appropriate safety protocols (e.g., social distancing, proper room ventilation). In some cases, video-led exercise may not be allowed on site due to COVID-19 restrictions, and in these instances the in-person group sessions include a 10-minute discussion about how to incorporate exercise into one’s daily routine. The coach will recommend daily physical activity for at least 20 minutes, whether in an ACHIEVE-D session or not, specifically emphasizing low impact moderate intensity aerobic exercises such as going on walks. In addition, video-based virtual exercise classes will be offered as a separate group, which will be facilitated by the study team’s peer leader and study staff. The peer leader is trained to co-lead these groups using a low-impact moderate intensity aerobic video (e.g., Walk Away the Pounds). When sites are delivering in-person groups and are not able to incorporate group exercise into the curriculum (due to COVID-19 safety protocols), these virtual exercise classes provide an option for clients to engage in exercise from the safety and convenience of their home while also benefiting from the support of their peers. To ensure participant safety during virtually delivered exercise groups, the study team’s technical support person will confirm each participants’ contact information at the beginning of each class (e.g., name, address from which they are joining the exercise class, phone number and emergency contact). The coach or peer leader will facilitate the class, and the study team member will monitor participants via video for safety.

*Weigh-In Component – 10 Minutes.* Next is the weigh-in section of group session during which coaches will review consumers’ progress towards previously set behavioral goals and provide feedback about changes in weight. Weight graphs will be utilized to provide a visual representation of consumers’ weight loss progress. Clients are encouraged to weigh-in during every class. Coaches will use motivational interviewing communication techniques when interacting with enrolled clients.

| **Appendix Table 2. Content of ACHIEVE-D Components** | | |
| --- | --- | --- |
| **Component** | **Contents** | |
| **Exercise** | Discuss the benefits of exercise and strategies to increase exercise participation (in-person)  Video-assisted group exercise (remote and as permitted in-person per COVID-19 restrictions) | |
| **Core modules** | Module 1: Weight loss success  Module 2: No sugar drinks  Module 3: No junk food | Module 4: Eat smart portions  Module 5: Eat more vegetables  Module 6: Putting it all together |
| **Seasonal modules** | A new start in the New Year  Heart healthy Valentine’s Day  Spring into walking | Maintain don’t gain during celebrations  Halloween fun without the sugar  Maintain don’t gain during the holidays |
| **Key behavioral recommendations** | Say no to sugar drinks  Say no to junk food  Say yes to eat smart portions | Say yes to eat more vegetables  Exercise for 20 minutes |

*Weight Management Component – 20 Minutes.* The coach will then review the main weight loss topic for the week. The coach reinforces the lesson topic with posters, handouts, and role model videos: scripted educational videos designed to provide examples of how peers are applying the recommended ACHIEVE-D behavioral strategies. The ACHIEVE-D curriculum includes six core monthly modules with each week of the module focusing on a specific lesson topic. The first module, Weight Loss Success, focuses on fundamentals of the program and the mechanism for weight loss (e.g., energy balance and tracking for self-awareness) to help consumers initiate safe and effective weight loss. In Modules 2 – 5, we will focus on key behavioral strategies for weight loss (No Sugar Drinks, No Junk Food, Eat Smart Portions, and Eat More Vegetables); each week within the module addresses implementing the behavioral strategy from a different perspective (e.g., while at the program, while at home, when eating out). The last module, Putting It All Together, summarizes key messages and focuses on building consumers’ confidence in maintaining healthy eating and exercise behaviors after the program ends. When more than one session is held within a given week, the lesson content is repeated to help consumers learn the key concepts and build self-efficacy to make changes; repetition is a key tenet of intervention tailoring for adults with SMI.^[[8]](#footnote-8)^ Additionally, six seasonal modules (e.g., Heart Healthy Valentine’s Day, Maintain Don’t Gain during the Holidays) are incorporated into the curriculum to help consumers apply the core behaviors during times of year when it may be challenging to maintain healthy eating and exercise behaviors.

*Goal Setting Component – 10 minutes.* Each group concludes with individualized goal setting; consumers will identify specific eating and exercise behaviors to work on between lessons that will lead to weight loss. Consumers will also be encouraged to track adherence to the ACHIEVE-D core behaviors using a simplified tracker to increase self-awareness and identify eating and exercise patterns. Finally, the curriculum encourages a goal of a 5% weight loss with an initial focus on the first 5 pounds.

*After the Group Session.* Coaches will document consumers’ attendance at group session, participation in exercise (during remotely delivered lessons), current weight, and high impact behavioral goals.

*Adaptations due to COVID-19 Pandemic.* Based on discussions with and requests from the study sites, each site will decide how they will deliver the ACHIEVE-D curriculum during the COVID-19 pandemic, taking into consideration how they are delivering their other programming, their priorities, and resources. Sites follow Maryland Medicaid and other regulatory guidelines for safety and operating procedures. Sites will have the flexibility to change their delivery method during the study. If programs are delivering in-person programming, then they will likely plan to deliver ACHIEVE-D in-person at least once per week. For persons with SMI, in-person programming is preferred due to psychiatric symptoms, cognitive impairment, and potential challenges using smartphones and computers. To support sites that prefer to offer remote groups, the study will be providing clients with easy-to-use tablets that include a data plan (Grandpads). Our study team will train enrolled clients to use the devices during baseline data collection and coaches will troubleshoot technology issues that arise during group sessions.

1. Daumit GL, Dickerson FB, Wang NY, Dalcin A, Jerome GJ, Anderson CAM, et al. A Behavioral Weight Loss Intervention in Persons with Serious Mental Illness. N Engl J Med. 2013;368:1594-602 [↑](#footnote-ref-1)
2. Bandura A. Social foundations of thought and action: a social cognitive theory. Englewood Cliffs, NJ: Prentice-Hall, Inc.; 1986. [↑](#footnote-ref-2)
3. Heinssen RK. The cognitive exoskeleton: Environmental interventions in cognitive rehabilitation. 1996. [↑](#footnote-ref-3)
4. Mueser KT, Corrigan PW, Hilton DW, Tanzman B, Schaub A, Gingerich S, et al. Illness management and recovery: a review of the research. Psychiatric services. 2002;53(10):1272-84. [↑](#footnote-ref-4)
5. Elmer PJ, Obarzanek E, Vollmer WM, Simons-Morton D, Stevens VJ, Young DR, et al. Effects of comprehensive lifestyle modification on diet, weight, physical fitness, and blood pressure control: 18-month results of a randomized trial. Annals of internal medicine. 2006;144(7):485-95. [↑](#footnote-ref-5)
6. Whelton PK, Appel LJ, Espeland MA, Applegate WB, Ettinger Jr WH, Kostis JB, et al. Sodium reduction and weight loss in the treatment of hypertension in older persons: a randomized controlled trial of nonpharmacologic interventions in the elderly (TONE). Jama. 1998;279(11):839-46. [↑](#footnote-ref-6)
7. Jerome GJ, Dalcin AT, Young DR, Stewart KJ, Crum RM, Latkin C, et al. Rationale, design and baseline data for the Activating Consumers to Exercise through Peer Support (ACE trial): A randomized controlled trial to increase fitness among adults with mental illness. Mental health and physical activity. 2012;5(2):166-74 [↑](#footnote-ref-7)
8. Cabassa LJ, Ezell JM, Lewis-Fernández R. Lifestyle interventions for adults with serious mental illness: a systematic literature review. Psychiatric services. 2010;61(8):774-82. [↑](#footnote-ref-8)
