# Supplementary material for: Comparing Implementation Strategies for an Evidence-Based Weight Management Program Delivered in Community Mental Health Programs: Protocol for a Pilot Randomized Controlled Trial
Source: JMIR Res Protoc. 2023 May 10;12:e45802. doi: 10.2196/45802 (PMC10209790; doi:10.2196/45802)
Supplement: Multimedia Appendix 3 [file resprot_v12i1e45802_app3.docx]

**Multimedia Appendix** **3: Other Planned Measures for Coaches and Individuals with Serious Mental Illness**

Coaches

In addition to the primary measures for coaches described in the main document, the following additional measures will be collected. Appendix Table 3.1 describes the assessment schedule for all other measures for coaches.

| **Appendix Table 3.1. Data Collection Schedule of Other Measures For Coaches** | | | |
| --- | --- | --- | --- |
|  | **Baseline** | **Follow-up** | |
|  |  | **During** | **6 Months** |
| ***Other Measures*** |  |  |  |
| Demographics & prior training | 🗸 |  |  |
| Attitudes towards individuals with obesity | 🗸 |  |  |
| Body mass index from self-reported height & weight | 🗸 |  | 🗸 |
| CARDIA/EARLY Q – sedentary behavior | 🗸 |  | 🗸 |
| Block Dietary Fat Q | 🗸 |  | 🗸 |
| Block Dietary Fruit/Vegetable Q | 🗸 |  | 🗸 |
| EARLY Eating Away from Home Q | 🗸 |  | 🗸 |
| EARLY SSB Consumption Q | 🗸 |  | 🗸 |
| Perceived Stress Scale | 🗸 |  | 🗸 |
| Self-Efficacy recall |  |  | 🗸 |
| *Abbreviations: Q – questionnaire; SSB – sugar-sweetened beverage.* | | | |

Other Measures

*Demographics and prior training.* We will collect demographic information such as age, sex, race and ethnicity. We will assess educational level and employment status. We will determine prior training in nutrition, exercise and weight loss at baseline, as well as their proficiency in using computers and online education. We will assess this at baseline, as prior training in these areas may influence our primary outcomes.

*Attitudes towards individuals with obesity*. We will determine attitudes towards individuals with obesity using elements from a previously validated measure.^[[1]](#footnote-1)^ We will assess this at baseline, as attitudes towards individuals with obesity may influence our primary outcomes.

*Sedentary behavior.* We will administer the CARDIA-EARLY Sedentary Behavior questionnaire, which is the measure recommended by the NIH for this outcome within weight loss trials.^[[2]](#footnote-2)^

*Dietary change.* We will administer Block Fat, Fruit, Vegetable and Fiber Screener Questionnaires as the food frequency measures.^[[3]](#footnote-3),^^[[4]](#footnote-4)^ These screeners provide a reasonably valid assessment of intake of these foods and have the advantage of being very brief. We will also administer the EARLY Eating Away from Home and Sugar-Sweetened Beverage questionnaires, which are the measures recommended by the NIH for these outcomes within weight loss trials.^[[5]](#footnote-5)^

*Stress.* We will administer the perceived stress scale,^[[6]](#footnote-6)^ which is the measure recommended by the NIH for this outcome within weight loss trials.

*Self-efficacy recall.* Given that the primary self-efficacy measure may be subject to response shift bias,^[[7]](#footnote-7),^^[[8]](#footnote-8)^ we will also have coaches reflect upon their baseline ability to carry out these elements at the 6-month point, which will enable us to determine whether response shift bias may be present. This instrument asks participants to recall their self-efficacy before training.

Individuals with Serious Mental Illness (SMI)

In addition to the secondary outcome measures for patients with SMI described in the main document, the following additional measures will be collected. We will use several measures to assist in determining eligibility for the study. Appendix Table 3.2 describes the assessment schedule for all other measures for individuals with SMI, including screening.

| **Appendix Table 3.2. Data Collection Schedule of Other Measures for Individuals with SMI** | | | |  |
| --- | --- | --- | --- | --- |
|  | **Screening/Baseline** | **Follow-up** | |  |
|  |  | **During** | **6 Months** |  |
| Demographics | B |  |  |  |
| Exercise pre-participation screening | S |  |  |  |
| Medical history | S |  |  |  |
| Medications | S |  | 🗸 |  |
| Readiness rulers | S |  |  |  |
| Measured height | S |  |  |  |
| Body mass index | S |  | 🗸 |  |
| Blood pressure | S |  |  |  |
| NHIS 5-Factor Dietary Screener | B |  | 🗸 |  |
| Palatable Eating Motives Scale – Coping Subscale | B |  | 🗸 |  |
| Reward-based Eating Drive Scale | B |  | 🗸 |  |
| Perceived Stress Scale | B |  | 🗸 |  |
| Important Others Q | B |  | 🗸 |  |
| Medical Events |  |  | 🗸 |  |
| *Abbreviations: B – baseline; NHIS – National Health Interview Survey; Q – questionnaire; S – screening.* | | | | |

Other Measures

*Demographics.* We will collect demographic information such as age, sex, race, and ethnicity. We will also assess how often they attend the program.

*Exercise pre-participation screening (modified 2018 PAR-Q):* This modified physical activity readiness questionnaire will be used to assess if consumer is able to participate in exercise classes.

*Medical History:* We will collect medical history information from each consumer to assess ability to participate in the ACHIEVE program and the staff.

*Medications:* We will list all of the consumer’s current medications.

*Readiness Rulers:* We will use this tool to assess participant’s readiness to lose weight and willingness to participate in the ACHIEVE program.

*Anthropometrics – blood pressure, height and body mass index (BMI)*. Blood pressure will be determined by the OMRON 907 XL, a validated device that records BP using an oscillometric technique.^[[9]](#footnote-9)^ One blood pressure with 3 measurements (each separated by 30 seconds) will be obtained on the right arm of participants after they rest quietly in the seated position for at least 15 minutes.^[[10]](#footnote-10)^ If measures cannot be in person, participants will self-measure weight on a scale the team provides for them. Height will be measured to the nearest 0.1 cm using a wall-mounted stadiometer. Measured height and weight will be used for calculation of BMI, calculated as the Quetelet index (kg/m^2^).

*Dietary Screening.* We will administer the NHIS 5-Factor Dietary Screener Questionnaire with 26 questions.^[[11]](#footnote-11)^

*Eating habits.* We will administer two measures -- Palatable Eating Motives Scale (PEMS) – Coping Subscale^[[12]](#footnote-12)^ and Reward-based Eating Drive (RED) Scale,^[[13]](#footnote-13)^ which assess emotional factors that influence eating habits. We will also administer the Important Others Questionnaire that assess social factors that influence eating habits. All of these measures recommended by the NIH for these outcomes within weight loss trials.

*Stress.* We will administer the perceived stress scale,^14^ which is the measure recommended by the NIH for this outcome within weight loss trials.

*Important others.* We will capture information about support systems and people in the consumer’s life that can assist in healthy weight management behaviors.^[[14]](#footnote-14)^

*Medical events.*  We will also capture any medical events that occur during the 6-month intervention.

1. Foster GD, Wadden TA, Makris AP, et al. Primary Care Physicians’ Attitudes about Obesity and Its Treatment. Obesity Research 2003;11:1168-77 [↑](#footnote-ref-1)
2. Gibbs BB, King WC, Davis KK, Rickman AD, Rogers RJ, Wahed A, et al. Objective vs. Self-report Sedentary Behavior in Overweight and Obese Young Adults. J Phys Act Health. 2015; 12: 1551–7. [↑](#footnote-ref-2)
3. Block G, Clifford C, Naughton MD, Henderson M, McAdams M. A brief dietary screen for high fat intake. J Nutr Educ. 1989;21:199-207 [↑](#footnote-ref-3)
4. Block G, Gillespie C, Rosenbaum EH, Jenson C. A rapid food screener to assess fat and fruit and vegetable intake. Am J Prev Med. 2000;18:284-8. [↑](#footnote-ref-4)
5. Nelson MC, Lytle LA. Development and evaluation of a brief screener to estimate fast-food and beverage consumption among adolescents. J Am Diet Assoc. 2009; 109:730-4. [↑](#footnote-ref-5)
6. Cohen S, Kamarck T, Mermelstein R. A global measure of perceived stress. J Health Soc Behav 1983;24:385-96. [↑](#footnote-ref-6)
7. Manthei RJ. The response-shift bias in a counsellor education programme. British Journal of Guidance and Counselling 1997;25:229-37 [↑](#footnote-ref-7)
8. Howard GS. Response-shift bias: A problem in evaluating interventions with pre/post self-reports. Evaluation Review 1980;4:93-106 [↑](#footnote-ref-8)
9. White WB, Anwar YA. Evaluation of the overall efficacy of the Omron office digital blood pressure HEM-907 monitor in adults. Blood Press Monit 2001;6:107-10. [↑](#footnote-ref-9)
10. Vollmer WM, Appel LJ, Svetkey LP, et al. Comparing office-based and ambulatory blood pressure monitoring in clinical trials. J Hum Hypertens 2005;19:77-82. [↑](#footnote-ref-10)
11. National Cancer Institute. (2005). Five-factor screener in the 2005 National Health Interview Survey cancer control supplement. Retrieved from <http://epi.grants.cancer.gov/nhis/5factor/> [↑](#footnote-ref-11)
12. Boggiano MM, Wenger LE, Turan B, et al. Real-time sampling of reasons for hedonic food consumption: further validation of the Palatable Eating Motives Scale. Front Psychol. 2015;6:744. [↑](#footnote-ref-12)
13. Epel ES, Tomiyama AJ, Mason AE, et al. The Reward-Based Eating Drive Scale: A Self-Report Index of Reward-Based Eating. PLoS ONE. 2014; 9:e101350. [↑](#footnote-ref-13)
14. Williams GC, Lynch MF, McGregor HA, Ryan RM, Sharp D, Deci EL. Validation of the "Important Other" Climate Questionnaire: Assessing autonomy support for health-related change. Families, Systems, & Health 2006;24:179-94. [↑](#footnote-ref-14)
